# Long-Term L-Glutamine Treatment Reduces Hemolysis without Ameliorating Hepatic Vaso-Occlusion and Liver Fibrosis in a Mouse Model of Sickle Cell Disease

**DOI:** 10.3390/biomedicines11092412

**Published:** 2023-08-29

**Authors:** Omika Katoch, Ramakrishna Ungalara, Tomasz Kaminski, Ziming Li, Rikesh K. Dubey, Isabella Burholt, Shweta Gudapati, Tirthadipa Pradhan-Sundd

**Affiliations:** 1 Pittsburgh Heart, Lung and Blood Vascular Medicine Institute, University of Pittsburgh School of Medicine, Pittsburgh, PA 15261, USA; 2 Division of Hematology/Oncology, Department of Medicine, University of Pittsburgh School of Medicine, Pittsburgh, PA 15261, USA

**Keywords:** sickle cell disease, hemolysis, vaso-occlusion, L-glutamine, liver injury, Kupffer cells

## Abstract

Sickle cell disease (SCD) is an autosomal recessive monogenic disorder caused by a homozygous mutation in the β-globin gene, which leads to erythrocyte sickling, hemolysis, vaso-occlusion, and sterile inflammation. The administration of oral L-glutamine has been shown to reduce the frequency of pain in SCD patients; however, the long-term effect of L-glutamine in SCD remains to be determined. To understand the long-term effect of L-glutamine administration in the liver we used quantitative liver intravital microscopy and biochemical analysis in humanized SCD mice. We here show that chronic L-glutamine administration reduces hepatic hemoglobin–heme–iron levels but fails to ameliorate ischemic liver injury. Remarkably, we found that this failure in the resolution of hepatobiliary injury and persistent liver fibrosis is associated with the reduced expression of hepatic Kupffer cells post-L-glutamine treatment. These findings establish the importance of investigating the long-term effects of L-glutamine therapy on liver pathophysiology in SCD patients.

## 1. Introduction

Sickle cell disease (SCD) is an autosomal recessive monogenic disorder with an estimated annual medical cost over USD 1.1 billion in the US [1,2,3,4]. A point mutation at the sixth position in β-globin gene substituting glutamic acid with valine results in sickled hemoglobin (HbS) [4]. Patients homozygous for this mutation are at increased risk for developing multiple organ failure due to vaso-occlusion, hemolysis, and sterile inflammation [4]. Liver abnormalities in SCD are frequent and the disease etiology remains largely unknown [5,6,7]. Elevated levels of liver enzymes (alanine aminotransferase (ALT), aspartate aminotransferase (AST), alkaline phosphatase (ALP)), hepatic iron–heme–hemoglobin accumulation, inflammation, and abnormal coagulation are commonly seen in hospitalised SCD patients [8,9,10,11,12]. Currently, there are no effective medical therapies available for SCD related liver problems, and the management of the disease is mostly limited to supportive therapy [13,14,15].

L-glutamine was approved by the U.S. Food and Drug Administration (FDA) for use in sickle cell disease (SCD) patients in 2017. L-glutamine is a conditionally essential amino acid required for the synthesis of nicotinamide adenine dinucleotide (NAD), glutathione and glutamate, and reduces oxidative stress [16]. Previous studies have shown that L-glutamine administration protects against the adhesion of sickle red blood cells in patients [17,18,19]. In a randomized, double-blind, controlled trial, L-glutamine ameliorated episodes of pain crisis in children and adults [19]. However, L-glutamine treatment was also associated with a few limitations, such as low toleration (only tolerated in two-thirds of patients) as well as organ complications [20,21]. To understand the effect of L-glutamine in SCD-related liver dysfunction, we evaluated the long-term effect of L-glutamine administration in the livers of SCD patients. Here, we show that eight weeks of L-glutamine treatment in SCD mice significantly reduced the accumulation of hemoglobin–heme–iron without ameliorating ischemic liver injury and fibrosis in SCD mouse liver. Remarkably, we find that this failure in the resolution of hepatobiliary injury post-L-glutamine treatment is associated with the reduced expression of hepatic Kupffer cells.

## 2. Methods

Animals: Townes SCD mice (SS, homozygous for Hba^tm1(HBA)Tow^, homozygous for Hbb^tm2(HBG1,HBB*)Tow^) and non-sickle control mice (AS, homozygous for Hba^tm1(HBA)Tow^, compound heterozygous for Hbb^tm2(HBG1,HBB*)Tow^/Hbb^tm3(HBG1,HBB)Tow^) [22] were obtained from the Jackson Laboratory (Bar Harbor, ME, USA) and housed in a specific pathogen-free animal facility at the University of Pittsburgh. All animal experiments were approved by the Institutional Animal Care and Use Committee at the University of Pittsburgh. In total, 3–5 mice were assessed at all given time points. 

L-glutamine treatment: Briefly, 5–6-week-old SCD mice received L-glutamine treatment via drinking water at a dosage of 10–12 mg/mL [23].

Surgical preparation and quantitative liver intravital imaging (qLIM): Details of the surgical method are described here [24,25]. Intravascular fluorescent dyes included 200 μg of Texas red (TXR) dextran which was used to visualize the blood flow through the liver sinusoids. Microscopy was performed using a Nikon MPE multi-photon excitation microscope at CBI U.Pitt. The percentage of regions with vaso-occlusion (as seen by TXR-Dextran staining) per field of view (FOV) was quantified from at least 3 different mice/group. 

Iron colorimetric assay: Hepatic total iron and Fe^2+^ and Fe^3+^ levels were measured using an iron colorimetric assay, as per the manufacturer’s instructions (Iron Assay Kit, ABCAM, ab83366). Briefly, liver tissue was homogenized in PBS. The homogenates were centrifuged at 6000× *g* for 10 min to remove debris. Iron levels were measured, and concentrations were determined using the calibration curve and mean change in the absorbance value for each sample. 

Heme assay: A heme assay was performed as per the manufacturer’s instructions (Heme assay kit; ABCAM; ab272534). Briefly, liver tissue was homogenized as directed. The homogenates were centrifuged, and heme levels were measured for each sample as per the manufacturer’s instructions.

Immunohistochemistry. Tissue samples were frozen in OCT compound (Sakura, 4583) on dry ice and stored at −80 °C. Cryopreserved samples were cut into 5 µm sections, washed in PBS, and then fixed in 2% paraformaldehyde for 30 min. Following washing, the slides were washed with PBS and permeabilized with 0.1% Triton X-100 in PBS for 20 min at room temperature. The samples were washed three times with PBS and then blocked with 2% goat serum in 0.1% Tween-20 in PBS (PBST) for 30 min at room temperature. Antibodies were diluted in 2% goat serum/PBST and incubated at 4 °C overnight. The primary antibodies used include: F4/80 (CST, 70076S, 0.435 μg/mL), CLEF4C (R&D Systems, AF2784, 0.025 μg/mL) and (abcam, AB75973, 0.07 µg/mL). The secondary antibodies used include anti-Mouse/Rabbit Cy3/Cy5. Images were taken on a Nikon A1 spectral confocal microscope. 

mRNA isolation and real time polymerase chain reaction: mRNA was isolated and purified from the livers of SCD mice at the baseline and post-L-glutamine treatment (*n* = 4–5/group). mRNA was isolated using Trizol (Invitrogen, Waltham, MA, USA). RT-PCR was performed as described elsewhere [26]. 18S and GAPDH were used to normalize the m-RNA expression data. The sequences of the primers are as follows: 

CD45: F-GAACATGCTGCCAATGGTCT R-TGTCCCACATGACTCCTTTCC; 

F4/80: F-GCCCAGGAGTGGAATGTCAA R-CAGACACTCATCAACATCTGCG; 

IL1β: F-CCATGGCACATTCTGTTCAAA R-GCCCATCAGAGGCAAGGA; 

CLEC4F: F-GGAAAGTCATTCCAGACCCA R- AAGACGCCATTTAACCCACA; 

TGFβ: F-GTGTGGAGCAACATGTGGAACTCTA R-TTGGTTCAGCCACTGCCGTA; 

α-SMA: F-GTTCAGTGGTGCCTCTGTCA R-ACTGGGACGACAGGAAAAG; 

Col1A1: F-TAAGGGTACCGATGGAGAAC R-CTCCCTGAGCTCCAGCTTCT; 

Col3A1: F-TCCCCTGGAATCTGTGAATC R-TGAGTCGAATTGGGGAGAAT; 

HBA1: F-ACTCTTCTGGTCCCCACAGACTCAG R-GGGCAGAGCCGTGGCTCAGGTCGAA; 

ACS14: F-CGTTTGGCTCATGTGCTGGAAC R-AGTCCAGGGATACGTTCACAC; 

PTGS2: F-GGGAGTCTGGAACATTGTGAA R-GTGCACATTGTAAGTAGGTGGACT; 

GAPDH: F-GACAGTCAGCCGCATCTTCT R-TTAAAAGCAGCCCTGGTGAC; 

18S: FCGGCTACCACATCCAAGGAA R-GCTGGAATTACCGCGGCT. 

Western Blot: Immunoblotting was performed as described elsewhere [27]. The primary antibodies used in this study include: CLEC4F (R&D Systems, AF2784, 0.025 μg/mL), CD45 (CST, 70257S, 19 µg/mL, HbA1 (abcam, Cambridge, UK, AB92492, 0.815 mg/mL), HbA2 (ABclonal, Woburn, MA, USA, A8427, 1.203 µg/mL), and Ferritin (abcam, AB75973, 0.07 µg/mL). The membranes were washed four times for 5 m each in TBST before being probed with HRP-conjugated secondary antibodies (1:5000 diluted in TBST; Santa Cruz Biotechnology, Dallas, TX, USA)/IgG conjugates secondary antibodies (1:5000/1:30000 diluted in TBST; Santa Cruz Biotechnology) for 1.5 h at room temperature. The membranes were washed three times for 10 m each in TBST and visualized using the Enhanced Chemiluminescence System (GE Healthcare, Chicago, IL, USA)/Odyssey Clx li-cor system. 

Statistical Analysis: All comparisons between two groups were deemed statistically significant by an unpaired two-tailed Student’s *t*-test if *p* < 0.05. (*) denotes *p* < 0.05.

Serum biochemistry: Aspartate aminotransferase (AST) and alanine aminotransferase (ALT) were measured in serum samples taken before sacrifice. Serum biochemistry was measured by automated testing in the Clinical Chemistry Division, University of Pittsburgh School of Medicine.

## 3. Results and Discussion

**Figure 1:**
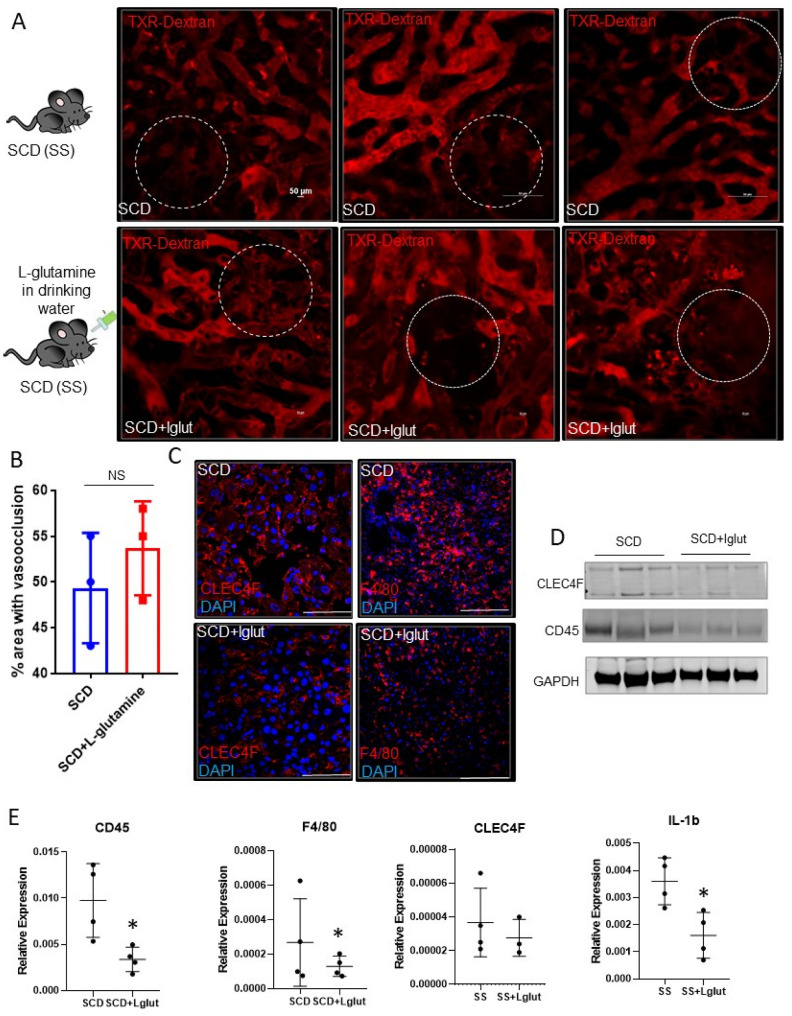
L-glutamine treatment does not ameliorate ischemic injury in SCD mouse liver. (**A**) Quantitative liver intravital (qLIM) imaging of three different fields of view of SCD mice at baseline and post-L-glutamine administration injected with TXR-dextran. Dotted circle shows loss of blood flow in SCD liver which was comparable in L-glutamine-treated SCD mouse liver. (**B**) Quantification of the total area (%) of liver with loss of blood flow in SCD mice at baseline and post-L-glutamine treatment. (**C**) Representative IF images show enhanced CLEC4F and F4/80 expression in SCD liver which was not seen after L-glutamine administration. (**D**) Western Blot for CLEC4F and CD45 antibodies exhibits increased expression in the liver of SCD mice compared to L-glutamine-treated SCD mouse liver. (**E**) qRT-PCR analysis exhibits reduced mRNA expression of markers of inflammatory cells (including F4/80, Cd45, CLEC4F, and IL1β) in L-glutamine-treated SCD liver compared to SCD liver at baseline. * denotes *p* < 0.05, NS, Not significant.

Sinusoidal vaso-occlusion is a common phenotype associated with SCD [4]. Previously, we have shown that SCD (SS) mice manifest sinusoidal ischemia and hepatobiliary injury under baseline conditions [7]. Here, we administered L-glutamine for up to eight weeks in SCD mice to study its effect on hepatic blood flow. Identical to our previous findings, quantitative liver intravital imaging (qLIM) revealed sinusoidal ischemia in several regions of the liver in SCD mice at the baseline (Figure 1A; upper panel; Supplemental Videos S1–S3). As shown in Figure 1A, these ischemic areas were evident as black voids in qLIM images due to the absence of TXR-dextran (red), suggestive of blood flow stasis. Interestingly, the blood flow stasis (red) did not show significant amelioration within the sinusoids of L-glutamine-treated SCD mice (*n* = 3; Figure 1A (lower panel), Supplemental Videos S4–S6). Further quantification confirmed that sinusoidal ischemia was comparable in the livers of L-glutamine-treated SCD mice (Figure 1B) and SCD mice at the baseline. Vaso-occlusion is associated with increased vascular cell adhesion and sterile inflammation [4,7]. Previously, we have shown the activation of inflammatory cells, including hepatic Kupffer cells, in the liver of SCD mouse at the baseline [7,28]. We next examined the Kupffer cell population post-L-glutamine treatment. SCD mice showed an enhanced expression of hepatic Kupffer cell markers CLEC4F and F4/80 staining, which were reduced in L-glutamine-treated SCD liver (Figure 1C). Western blot analysis showed a reduced expression of CLEC4F and CD45 protein in L-glutamine-treated SCD mouse liver (Figure 1D). Moreover, gene expression analysis of liver mRNA from the L-glutamine-treated SCD mice compared with SCD mice at the baseline showed a significant reduction in the expression of inflammatory cell markers (including F4/80, ClEC4F, CD45 and cytokines (IL1β)) (Figure 1E) post-L-glutamine treatment suggestive of reduced activation of inflammatory cells in SCD mouse liver post-L-glutamine treatment. 

**Figure 2:**
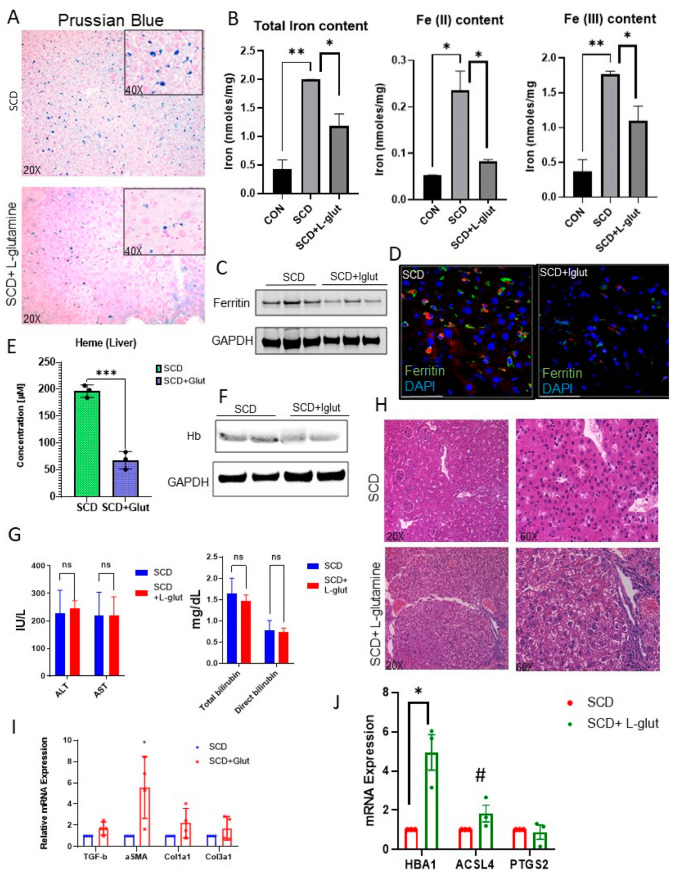
L-glutamine-treated SCD mice exhibit reduced hemolysis but fail to resolve chronic liver injury and fibrosis. (**A**) Prussian blue staining for iron showed increased iron deposition with a mixed distribution in hepatocytes and Kupffer cells in SCD liver which was mildly reduced in post-L-glutamine treatment. (**B**) Iron colorimetric assay exhibits the amount of total iron and Fe^2+^ and Fe^3+^ content in SCD mouse liver at baseline and post-L-glutamine treatment. (**C**) Western blot for ferritin exhibits increased expression in the liver of SCD mice compared to L-glutamine-treated SCD mouse liver. (**D**) Representative IF images showing Ferritin expression in SCD mouse liver at baseline and post-L-glutamine treatment. (**E**) ELISA assay shows significant reduction in hepatic heme levels post-L-glutamine treatment in SCD mouse liver compared to baseline heme level. (**F**) Western blot for hemoglobin exhibits increased expression in the liver of SCD mice compared to L-glutamine-treated SCD mouse liver. (**G**) Serum ALT, AST, and direct and total bilirubin levels in SCD mice at baseline and post-L-glutamine treatment. (**H**) H&E staining of SCD and L-glutamine-treated liver sections revealed increased fibrosis post-L-glutamine treatment. (**I**) Analysis of mRNA expression by qRT-PCR showed increase in mRNA expression of TGFβ, αSMA, Col1A1, and Col3A1 in L-glutamine-treated SCD mouse liver compared to baseline. (**J**) Analysis of mRNA expression by qRT-PCR showed increase in mRNA expression of cell death–ferroptosis and fibrosis markers (HBA1, ACSL4, and PTGS2) in L-glutamine-treated SCD mouse liver compared to baseline. * denotes *p* < 0.05, ** *p* < 0.01, *** p < 0.005, # *p <* 0.07, ns, Non significant.

Kupffer cells facilitate hepatic iron–heme–hemoglobin recycling [29,30,31]. As we see the reduced expression of Kupffer cells in L-glutamine-treated SCD liver, we examined hemoglobin–heme–iron accumulation in SCD mouse liver. Prussian blue staining revealed hepatic iron accumulation is SCD liver (Figure 2A), which was mildly reduced in L-glutamine-treated SCD liver. We next determined the hepatic iron level by performing an iron colorimetric assay. As shown in Figure 2B, L-glutamine administration resulted in a reduction in total iron and Fe^2+^ and Fe^3+^ content in SCD mouse liver. When examined, we found an overall reduction in ferritin expression, the surrogate marker for iron accumulation, in L-glutamine-treated SCD mouse liver via Western blot (Figure 2C) and immunofluorescence (Figure 2D) analysis. Similarly, we found a significant reduction in the hepatic heme (Figure 2E) and hemoglobin (Figure 2F) level, as detected by ELISA and Western blot analysis, respectively, in L-glutamine-treated SCD mice compared to SCD mice with no treatment. Interestingly, serum markers of liver injury (ALT and AST) did not show any improvement post-L-glutamine treatment compared to their baseline values in SCD mouse (Figure 2G). In addition, we found sinusoidal congestion and ballooning of cells by H&E staining (Figure 2H) post-L-glutamine treatment in SCD liver. qRT-PCR analysis confirmed the significant upregulation of fibrosis markers (including TGFβ, αSMA, Col1A1, Col3a1) in L-glutamine-treated SCD mice (Figure 2I) compared to untreated SCD mice. As we see unaltered vaso-occlusion and liver fibrosis in L-glutamine-treated SCD mice, we hypothesized that the ongoing liver fibrosis seen in L-glutamine-treated SCD mice is caused by the inefficient clearance of hepatic hemoglobin–heme–iron due to Kupffer cell depletion [31,32,33]. Remarkably, when analyzed, we found a significant increase in some of the liver-fibrosis- and cell-death-associated markers (such as HBA1, ACSL4, and PTGS2) in L-glutamine-treated SCD mouse liver compared to untreated SCD mice (Figure 2J). The observed increase in overall liver injury and activated myofibroblasts may also indicate the activation of hepatic stellate cells following L-Glutamine treatment, which should be investigated in future research. Taken together, these data suggest that long-term L-glutamine treatment can reduce hepatic heme–hemoglobin–iron level but has no effect on vaso-occlusion-associated acute ischemic injury and liver fibrosis in SCD. Moreover, long-term L-glutamine treatment depletes hepatic Kupffer cells, leading to fibrosis and hepatic cell death. 

Our current study is the first to highlight the long-term effects of L-glutamine in SCD mouse liver. Interestingly, despite a considerable decrease in hepatic heme–hemoglobin–iron accumulation, chronic liver injury did not ameliorate post-L-glutamine treatment. We hypothesize that this could be due to the following reasons. Firstly, the injury was already established in SCD mice prior to L-glutamine treatment. Thus, the reduced expression of hepatic Kupffer cells contributed to the impaired clearance of Hb-Heme–iron in the liver, leading to tissue damage. Secondly, the persistent injury and fibrosis seen in SCD mice post-L-glutamine treatment could be solely due to the ongoing vaso-occlusive crisis. Thirdly, increased hepatic cell death following L-glutamine treatment can also result in persistent liver fibrosis in SCD mice. 

Organ damage in SCD is caused by both hemolysis and vaso-occlusion [4]. In this study, we found that L-glutamine is potentially useful to reduce hemolysis but does not have any beneficial effect on attenuating hepatic vaso-occlusion. Emerging evidence emphasizes the significance of a combinatorial approach in addressing the multifaceted pathophysiology associated with SCD [34]. Our research suggests that one such combinatorial strategy could involve adding L-glutamine and inhibiting p-selectin simultaneously. Previously, we have shown that blocking p-selectin can significantly ameliorate vaso-occlusion without affecting hemolysis [28]. Therefore, future studies should investigate the simultaneous effect of L-glutamine administration and p-selectin inhibition in attenuating SCD-associated vaso-occlusion and hemolysis. 

A major limitation of our study is the use of L-glutamine in SCD mice to model the effects of L-glutamine therapy in SCD patients. Thus, species-specific differences cannot be ruled out. Also, in our current study, L-glutamine was administered for up to eight weeks via drinking water. L-glutamine therapy in SCD patients may not result in the same extent of liver pathophysiology as what was caused by the continuous administration in mice in our study. Notwithstanding these limitations, our current findings highlight the need to investigate the long-term effects of L-glutamine therapy on vaso-occlusion, Kupffer cell expression, hepatic cell death, and liver fibrosis in SCD patients. 

## Data Availability

All data related to this project is shared in this study.

## Supplementary Materials

The following supporting information can be downloaded at: https://www.mdpi.com/article/10.3390/biomedicines11092412/s1, Video S1: Visualization of blood flow in a SCD (SS) mouse after administration of TXR-dextran; Video S2: Visualization of blood flow in a SCD (SS) mouse after administration of TXR-dextran; Video S3: Visualization of blood flow in a SCD (SS) mouse after administration of TXR-dextran; Video S4: Visualization of blood flow in L-glutamine treated SCD mouse after administration of TXR-dextran; Video S5: Visualization of blood flow in L-glutamine treated SCD mouse after administration of TXR-dextran; Video S6: Visualization of blood flow in L-glutamine treated SCD mouse after administration of TXR-dextran.
