# Supplementary material for: Long-Term L-Glutamine Treatment Reduces Hemolysis without Ameliorating Hepatic Vaso-Occlusion and Liver Fibrosis in a Mouse Model of Sickle Cell Disease
Source: Biomedicines. 2023 Aug 29;11(9):2412. doi: 10.3390/biomedicines11092412 (PMC10526062; doi:10.3390/biomedicines11092412)
Supplement: Supplementary file 1 [file biomedicines-11-02412-s001.zip › biomedicines-2552897-supplementary.pdf]

Supplemental Material for

Long-term l-glutamine treatment reduces hemolysis without ameliorating hepatic vasoocclusion and liver fibrosis in a mouse model of sickle cell disease.

Omika Katoch[1,#], Ramakrishna Ungalara[1,#],Tomasz Kaminski[1,#], Ziming Li[1] , Rikesh K Dubey[1], Isabella Burholt[1], Shweta Gudapati[1], Tirthadipa Pradhan-Sundd[1,2,*]

1. Pittsburgh Heart, Lung and Blood Vascular Medicine Institute, University of Pittsburgh School of Medicine, Pittsburgh, PA, USA.
2. Division of Hematology/Oncology, Department of Medicine, University of Pittsburgh School of Medicine, Pittsburgh, PA, USA.

# these authors have contributed equally.

*To whom correspondence should be addressed:

Tirthadipa Pradhan-Sundd, PhD

Vascular Medicine Institute

Division of Hematology-Oncology

Department of Medicine

University of Pittsburgh School of Medicine

200 Lothrop Street, Pittsburgh, PA, 15261

Email: tip9@pitt.edu

Table of contents

Supplemental methods

Supplemental Movie Legends

Supplemental Methods

Heme assay: Heme assay was performed as per the manufacturer's instructions (Heme assay kit; ABCAM; ab272534). Briefly, liver tissue was homogenized as directed. The homogenates were centrifuged and heme levels were measured for each sample as per the manufacturer's instructions .

Immunohistochemistry . Tissue samples were frozen in OCT compound (Sakura, 4583) on dry ice and stored at - 80°C. Cryopreserved samples were cut into 5 μm sections, washed in PBS, and then fixed in 2% paraformaldehyde for 30 minutes. Following washing, slides were washed with PBS and permeabilized with 0.1% Triton X-100 in PBS for 20 minutes at room temperature. Samples were washed three times with PBS and then blocked with 2% goat serum in 0.1% Tween-20 in PBS (PBST) for 30 minutes at room temperature. Antibodies were diluted in 2% goat serum/PBST and incubated at 4°C overnight. Primary and secondary antibodies used are: Primary antibodies: F4/80 (CST, 70076S, 0.435 μg/ml), CLEF4C (R&D Systems, AF2784, 0.025μg/mL) and (abcam, AB75973, 0.07 μg/ml). Secondary antibodies used are anti-Mouse/Rabbit Cy3/Cy5. Images were taken on a Nikon A1 spectral confocal microscope.

mRNA isolation and real time polymerase chain reaction: mRNA was isolated and purified from livers of SCD mice at baseline and post l-glutamine treatment (n=4-5/group). mRNA was isolated using Trizol (Invitrogen). RT-PCR was performed as described elsewhere[27]. 18S and GAPDH were used to normalize the m-RNA expression data. Sequences of primers are as follows:

CD45: F-GAACATGCTGCCAATGGTCT R-TGTCCCACATGACTCCTTTCC;
F4/80: F- GCCCAGGAGTGGAATGTCAA R- CAGACACTCATCAACATCTGCG;
IL1β: F- CCATGGCACATTCTGTTCAAA R- GCCCATCAGAGGCAAGGA;
CLEC4F: F- GGAAAGTCATTCCAGACCCA R- AAGACGCCATTTAACCCACA;
TGFβ: F- GTGTGGAGCAACATGTGGAACTCTA R- TTGGTTCAGCCACTGCCGTA;
α-SMA: F- GTTCAGTGGTGCCTCTGTCA R- ACTGGGACGACAGGAAAAG;
Col1A1: F- TAAGGGTACCGATGGAGAAC R- CTCCCTGAGCTCCAGCTTCT;
Col3A1: F- TCCCCTGGAATCTGTGAATC R- TGAGTCGAATTGGGGAGAAT;
HBA1: F- ACTCTTCTGGTCCCCACAGACTCAG R- GGGCAGAGCCGTGGCTCAGGTCGAA;

ACS14: F- CGTTTGGCTCATGTGCTGGAAC R- AGTCCAGGGATACGTTCACAC;

PTGS2: F- GGGAGTCTGGAACATTGTGAA R- GTGCACATTGTAAGTAGGTGGACT;

GAPDH: F-GACAGTCAGCCGCATCTTCT R- TTAAAAGCAGCCCTGGTGAC;

18S: FCGGCTACCACATCCAAGGAA R- GCTGGAATTACCGCGGCT.

Supplemental Movie Supplemental Video Legends

Video S1. Visualization of blood flow in a SCD (SS) mouse after administration of TXR-dextran. The sinusoids in SCD mouse liver visualized by carotid artery injection of TXR-dextran (red). Here the blood flow is occluded at a region which appears dark due to lack of flow. Original acquisition rate. Scale bar 50 uM.

Video S2. Visualization of blood flow in a SCD (SS) mouse after administration of TXR-dextran. The sinusoids in SCD mouse liver visualized by carotid artery injection of TXR-dextran (red). Here the blood flow is occluded at various regions which appears dark due to lack of flow. Original acquisition rate. Scale bar 50 uM.

Video S3. Visualization of blood flow in a SCD (SS) mouse after administration of TXR-dextran. The sinusoids in SCD mouse liver visualized by carotid artery injection of TXR-dextran (red). Here the blood flow is occluded at regions which appears dark due to lack of flow. Original acquisition rate. Scale bar 50 uM.

Video S4. Visualization of blood flow in l-glutamine treated SCD mouse after administration of TXR- dextran. The sinusoids in l-glutamine treated SCD *mouse* liver visualized by carotid artery injection of TXR-dextran (red) and CF (green; as a marker for liver bile ducts). Here the blood flow appears continuous. Original acquisition rate. Scale bar 50 uM.

Video S5. Visualization of blood flow in l-glutamine treated SCD mouse after administration of TXR- dextran. The sinusoids in l-glutamine treated SCD *mouse* liver visualized by carotid artery

injection of TXR-dextran (red) and CF (green; as a marker for liver bile ducts). Here the blood flow appears continuous. Original acquisition rate. Scale bar 50 uM.

Video S6. Visualization of blood flow in l-glutamine treated SCD mouse after administration of TXR- dextran. The sinusoids in l-glutamine treated SCD *mouse* liver visualized by carotid artery injection of TXR-dextran (red) and CF (green; as a marker for liver bile ducts). Here the blood flow appears continuous. Original acquisition rate. Scale bar 50 uM.
